# Analysis of Parkinson’s Disease Outpatient Counselling for Advance Directive Creation: A Cross-Sectional Questionnaire-Based Survey of German General Practitioners and Neurologists

**DOI:** 10.3390/brainsci12060749

**Published:** 2022-06-07

**Authors:** Ida Jensen, Almut Bretschneider, Stephanie Stiel, Florian Wegner, Günter U. Höglinger, Martin Klietz

**Affiliations:** 1 Department of Neurology, Hannover Medical School, 30625 Hannover, Germany; almut.bretschneider@stud.mh-hannover.de (A.B.); wegner.florian@mh-hannover.de (F.W.); hoeglinger.guenter@mh-hannover.de (G.U.H.); klietz.martin@mh-hannover.de (M.K.); 2 Institute for General Practice and Palliative Care, Hannover Medical School, 30625 Hannover, Germany; stiel.stephanie@mh-hannover.de

**Keywords:** advance directives, palliative medicine, Parkinson’s disease, PwP, advance care planning

## Abstract

A major proportion of people with Parkinson’s disease (PwP) in Germany has written an advance directive (AD). Unfortunately, these ADs are unclear for PD-specific endpoints. We previously established consensus-based recommendations for disease-specific content of an AD in PwP. However, the implementation of those recommendations and the consulting of AD creation and modification in PwP remains to be evaluated. This study aimed to investigate the practical use of PD-specific recommendations for ADs in outpatient settings. A total of 87 physicians (45 general practitioners (GPs) and 42 neurologists, 10% response rate) answered a self-constructed semiquantitative questionnaire. The participants were asked to evaluate the suggested PD-specific recommendations for ADs and the supply of palliative care in the outpatient setting. Overall, the vast majority of treating physicians agreed on the usefulness of the newly constructed PD-specific recommendations. Consultations to discuss information about PD-specific ADs were scarce with short durations. Only 24% of participating physicians implemented the PD-specific recommendations in their daily practice. GPs and neurologists agreed on the benefit of disease-specific recommendations for ADs. In future, a more general integration of these recommendations in routine care might improve specific AD creation of PwP and advanced care planning.

## 1. Introduction

Parkinson’s disease (PD) is among the most common neurodegenerative diseases, affecting around 6.9 million people worldwide [1]. As no curative treatment has been found yet, the disease inevitably leads to progressing motor and non-motor symptoms [2]. Those symptoms can cause a loss of autonomy, forcing the treating physicians to adapt therapies regularly in order to meet the needs of people with PD (PwP) [3,4,5,6,7]. Further, physicians have to support PwP in planning future treatments in advance for when PwP become unable to express their wishes [8,9]. Mainly in advanced PD stages palliative care can be an option to improve the health-related quality of life and to reduce burden [10,11]. According to the WHO, palliative care is an approach to ease the suffering of patients and their families affected by a progressive illness due to the management of medical symptoms, psychosocial issues, spiritual concerns and planning for the future [12]. Palliative care concepts are well established in the context of oncological disorders [13,14]. As recent research displayed, patients with advanced neurological disorders can profit from palliative care as well [15].

Regarding PD, the benefit and increasing demand of palliative care has been demonstrated before [6,15,16,17,18]. One major aspect of palliative care is advance care planning. It involves education about diagnosis and prognosis, assignment of a health care power of attorney, and, in the optimal case, writing of a specific advance directive (AD) to identify goals, preferences and values of the patients [19]. In Germany and other western countries, ADs are essential instruments to improve medical decision making according to the patient’s will [6,16,20]. Previous research displayed that the majority of PwP in an advanced disease stage in Germany has a written AD [21]. However, those ADs covered mainly general aspects of care and were rather unspecific about PD-related symptoms and complications [21]. Unfortunately, no official guidance for writing a specific AD has been implemented yet [22,23]. To improve AD-creation of PwP, our group previously established consensus-based recommendations addressing PD-specific complications and therapy for implementation in ADs [5,21]. However, it has been unclear yet how treating physicians in an outpatient setting value those recommendations and whether those have been already implemented in clinical practice.

Therefore, we aimed to analyze the process of counselling AD-creation for PwP by German general practitioners (GPs) and neurologists in an outpatient setting. This study is the first to provide insights into the roll-out of PD-specific recommendations in ADs of PwP. The results will help to achieve a more efficient and personalized care for PwP in the future.

## 2. Materials and Methods

### 2.1. Ethics Approval

The study obtained approval from the local Ethics Committee of Hannover Medical School (No. 3123-2016, Amendment 2020). All health care professionals gave their informed consent to participate in this study.

### 2.2. Study Design

In this monocentric, cross-sectional, observational study the process of counselling an AD creation for PwP was analyzed. PD-specific recommendations for ADs concerning PD-related complications were evaluated by a group of neurologists and GPs by completing a questionnaire. These consensus-based recommendations were developed in a previous study of our research group [5]. Physicians were also asked to give their estimation to the current status quo concerning the palliative guidance of their patients with PD. The survey was conducted between March and May 2021, the procedure took about 10–15 min. There was no financial or other gratification for the participating physicians.

### 2.3. Development of the Questionnaire

The questionnaire was designed by MK, FW and SSt. The aim of the questionnaire was to survey GPs and neurologists about their opinion with regard to previously established AD recommendations and to get an overview about the current implementation of general palliative guidance of PwP in an outpatient setting.

The specifically designed questionnaire contains 33 questions to be answered using multiple choice and free texts format. It is composed of four parts. In the first part, the participants were asked about demographic aspects such as their age, sex, location of the practice and special training in palliative care. The second part comprised questions about their PwP like total number, the percentage of them in an advanced stage and the percentage of patients who obtain palliative care. We defined advanced stages of PD as a Hoehn and Yahr stage >3, presence of complex symptoms, severe non-motor symptoms or non-oral follow-up therapies [10,24]. The third part contains questions about ADs of PwP. The last and biggest part consisted of questions about the previously established recommendations for creation of a PD-specific AD.

### 2.4. Study Population

About 441 neurologists and 432 GPs in Lower Saxony were identified by the federal physician department (Landesärztekammer Niedersachsen). Neurologists and GPs were matched on a 1:1 basis by only addressing every tenth GP in the study. In total 873 questionnaires were sent to the selected physicians. Practices with urban and rural location were both included. The survey was done paper-based and the physicians were contacted by a letter including the questionnaire. As this survey was completely anonymous, there were no reminders for late or non-responders. 

### 2.5. Statistics 

Results of the survey were analyzed by mean and standard deviation. The following statistical tests were used when applicable: Linear Trend Test; Pearson Chi-Square Test; Fisher’s Exact Test; t-test; Pearson Chi-Square Test. 

Pearson Chi-Square Test, Fisher’s Exact Test and T-test were applied to analyze differences concerning demographic data of the survey participants. Linear Trend Test was applied to calculate significant differences of the answers concerning advance care planning in outpatient settings. To test for equality of variances before using a t-test the Levene’s test was used. Differences concerning the supply with PD-specific advance directives in outpatient settings were analyzed with Linear Trend Test, Pearson Chi-Square Test and Fisher’s Exact Test. Linear Trend Test and Pearson Chi-Square Test were applied to analyze differences concerning the evaluation of the previously established PD-specific recommendations. The statistical analysis was carried out using SPSS 25.0 (IBM, US, New York, Armonk).

## 3. Results

### 3.1. Demographics of Participating Physicians

87 physicians (45 GPs and 42 neurologists, 10% response rate) completed the questionnaires. The demographic data of the participating physicians are displayed in Table 1. The mean age was 53.5 (± 9) years and the majority of physicians was male (58.6%). The average work experience was 24 (± 9.3) years. No significant differences between GPs and neurologists allowed an effective matching of groups. GPs treated a higher number of patients per quarter (*p* < 0.001) and were more often located in rural areas than neurologists (*p* = 0.002). Further, more GPs (33.3%) received special palliative care training than neurologists (4.8%, *p* = 0.001).

### 3.2. Advance Care Planning of People with Parkinson’s Disease

General information about advance care planning of PwP are shown in Table 2. In comparison, neurologists treated significantly more PwP than the GPs did (*p* < 0.001). Also, the neurologists treated more PwP in advanced stages of the disease (*p* < 0.003). Regardless of their specialization, a majority of the physicians (57.7%) declared that none of their PwP or only 1–5% (35.6%) received palliative care.

### 3.3. Current Supply of PD-Specific Advance Directives

Physicians’ answers regarding the current supply with PD-specific ADs are shown in Table 3. In general, this supply was scarce, as 27.6% of physicians reported that less than 25% of their PwP had written an AD. In only 6.9% of the cases, more than 75% of their PwP were provided with a written AD. 19% of the neurologists did not know whether their PwP had written an AD or not. GPs more often counselled PwP to create or modify their AD (75.6%) than neurologists (54.8%, *p* 0.041). Further, GPs documented more frequently whether a written AD existed or not (GP 77.8%; neurologists 42.9%, *p* 0.003).

84% of GPs estimated that consultations concerning PD-specific ADs occurred 0–5 times in a year, 15.5% reported over 6 consultations a year. Most of the neurologists (69%) had 0–5 consultations for AD creation of PwP in a year and only 11.9% reported more than 10 annually. The duration of consultations differed, as the neurologists spent less time on these than the GPs (*p* 0.018).

One-third of the neurologists and 15.6% of the GPs included some of the previously published PD-specific recommendations in ADs, displaying a significant difference (*p* 0.03). Concerning the included PD-specific recommendations, the aspect addressed most often was “nutrition and airway management for swallowing disorders” (all = 25.3%), which was more often addressed by neurologists in comparison to GPs (*p* 0.023). “Dementia development and personality changes” was addressed by 23% of the physicians. “Catheterization for bladder and rectal disorders” was included in 17.2% of the cases, followed by “Levodopa carbidopa intestinal gel (LCIG) therapy” (5.7%), “Deep brain stimulation” (DBS) (2.3%) or “others” (3.4%). Concerning other important aspects, participants suggested for example the consideration of establishing a healthcare proxy, added immobility, the higher risk of pneumonia during virus pandemics, chronic pain syndrome and pain caused by rigor and akinesia. 

### 3.4. Evaluation of PD-Specific Recommendations for ADs

Participating physicians evaluated the relevance and usefulness of the previously established PD-specific recommendations for ADs. The results are displayed in Table 4. All participants considered early consultations to discuss and write a PD-specific AD as important in order to include PwP in the decision-making. Many physicians rated the provision of information on swallowing disorders and treatment options for bladder and rectal disorders as important (56.3%) or rather important (40.2%). Almost all neurologists (97.6%) and the majority of the GPs (75.6%) treated PwP with swallowing disorders (*p* 0.003). Almost all physicians (93.1%) could imagine advising their PwP on specific aspects of the therapy of swallowing disorders in the framework of an AD.

Most of the physicians agreed that early communication about possible neuropsychiatric symptoms and their treatment was important (56.3%) or rather important (28.7%). However, some participants stated that they did not want to scare or demoralize the PwP in the early course of the disease. All neurologists and 84.4% of the GPs treated PwP with neuropsychological symptoms. Consequently, almost all physicians (89.7%) could imagine including specific aspects of the therapy of neuropsychological symptoms in the written AD.

Concerning non-oral advanced therapies, 57.7% of the physicians considered the explanation of the value of a medical pump for a palliative care approach as (rather) important. Just 36.8% of physicians treated PwP with Levodopa Carbidopa Gastrointestinal Gel (LCIG) therapy. 69% could imagine to advise their PwP about specific aspects of the LCIG therapy when writing an AD. As only 40.2% of the physicians treated PwP with DBS (GPs 22.2%; neurologists 59.9%; *p* 0.001), less could imagine advising PwP about specific aspects of the DBS (58.6%).

Most of the physicians stated that it was generally possible to include the established PD-specific recommendations when writing the AD with the PwP (79.3%). 17% thought an implementation was impossible but named the economic framework or scarce time in the clinical routine as reasons not to proceed. The majority of the physicians (67.8%) agreed on a shared responsibility between GPs and neurologists concerning the advice on PD-specific ADs to make palliative care an interdisciplinary approach.

## 4. Discussion

Previous studies showed, that even though most of the PwP had written an AD in terms of general end-of-life aspects, the majority of those ADs was rather unspecific in regard to PD-associated endpoints [21]. In order to improve advance care planning of PwP, we recently established consensus-based PD-specific recommendations which could be implemented in ADs of PwP [5]. In this study, 87 physicians treating PwP in outpatient settings reported about their counselling of AD creation in PwP and evaluated those PD-specific recommendations. Our data suggest that the established recommendations were perceived as useful for the outpatient care setting.

However, the results display a discrepancy between the measured benefit of PD-specific recommendations and the actual implementation in clinical practice. PwP ADs were mostly unspecific and the recommendations rarely used by GPs and neurologists for several reasons. One common misconception is that palliative care is only applied at the end of life. Often, healthcare providers and PwP think advance care planning is not compatible with active disease management and do not address it [25,26]. Consistent with those observations, most physicians stated that none or only 1–5% of their PwP received palliative care. However, palliative care should be perceived as a chance to add a new layer of support for PwP and their caregivers [27]. Kluger et al. showed in their randomized clinical trial, including 584 people with PD or related disorders, that outpatient palliative care leads to a better disease outcome in comparison to treatment with standard care [28].

In this study, the major counselling of ADs was made by GPs. As they had received palliative care training more often, they might be more sensitized on the topic of ACP. GPs accompany more patients over a long time and regularly write ADs with them, so they might address the topic more frequently. However, the needs of PwP in the advanced disease stages are highly specific [21]. GPs are not expected to be skilled in the specific complications and needs of the vast variety of neurological diseases [29,30]. 

Most of the physicians stated that the appointment concerning writing ADs should be a shared approach between GP and neurologists. This reflects the general perception of palliative care as an interdisciplinary approach [31]. Collaboration and exchange between professions are crucial to constantly improve patient care [32,33,34]. Other specialists should be included in ACP of PwP as well. For example, our data suggest that specific recommendations concerning LCIG therapy and DBS have been hardly ever mentioned in the ADs. PwP often receive treatment concerning those specific therapies by specialists [35,36]. An interdisciplinary approach to inform PwP about all relevant complications of PD would be preferable to provide optimal support [19].

Nevertheless, our results reflect that neurologist should hold the primary responsibility in providing palliative guidance. As displayed in our data, the neurologists treated more PwP in advanced disease stages and included PD-specific recommendations more often, allowing them to develop a higher expertise about the needs of PwP.

Unfortunately, only about 5% of neurologists received palliative care training before. The lack of palliative care training for neurologists was reported previously [29]. Studies showed that neurologists without palliative care training tended to avoid the topic of palliative care. They felt uncomfortable talking about it or did not want to discourage the PwP [37,38]. As they did not receive special training, neurologists might also not be aware of palliative care opportunities to improve the care of PwP, as for example the established PD-specific recommendations for ADs.

However, Creutzfeldt et al. highlighted that neurologists should receive special palliative training, since they treat patients in advanced stages of neurologic diseases [39]. They should be prepared to discuss the palliative treatment options and prognosis of neurological illness with the patients and their relatives [39]. Further, our data revealed a lack of special training concerning technologies for treatment of PD. One example is deep brain stimulation (DBS), as it can change the axial symptoms predicting mortality and life expectancy of PwP [40]. Consequently, questions concerning ethical issues in palliative care and AD creation, such as new prominent disease trajectories, have to be discussed [41]. Here, neurologists have to receive specific training to address novel ethical issues caused by new treatments such as DBS for better engaging with AD.

Over the last years, several projects were established to increase neurological residents’ exposure to palliative care in their training [42,43,44]. Unfortunately, recent literature confirmed that economic shortages and a lack of workforce hinder sufficient training and care [45]. In this study, especially neurologists stated that consultations to discuss PD-specific ADs were scarce and short. There was little time left during the outpatient routine to address the issue of specific ADs. In another German cohort, half of the PwP in advanced stages did not even manage to get regular appointments with their neurologist [10].

Consequently, the economic and temporal framework has to be adapted to give physicians the chance to learn about palliative care and apply it in outpatient settings. Reimbursement policies for physicians consulting patients concerning their AD or in the context of advance care planning seem to be a promising intervention to increase the number and amount of time dedicated to these issues by the physicians. Also, this may motivate more neurologists to undergo specific palliative care training. Recently established AD recommendations can be used as guidance for discussion with their PwP. They may also allow to save time, as the important topics to discuss are already prepared and easy to apply in the outpatient setting. Overall, the PD-specific recommendations for ADs, when regularly implemented, could be a helpful instrument for physicians to improve the care of PwP in the future.

## 5. Limitations

In this cross-sectional questionnaire-based study, only physicians working in the German health care system were included. The examined AD recommendations are related to a middle European cultural background and may not easily be transferred to other cultures or societies. Similar investigations from different countries could help to overcome the limitations in generalizability. The physicians’ participation rate of 10% could indicate a ‘selection bias’ in data collection. However, we could depict a broad impression of the actual outpatient situation. Not all possible complications of PD were addressed in previous AD recommendations and this study. For example, PD-specific recommendations for ADs concerning complications of mobility, like falls, delirious symptoms or gait disorder, were not addressed. Those should be topics of future research.

## 6. Conclusions

The results highlight the usefulness of the previously established PD-specific recommendations for ADs in the context of outpatient care. However, these data demonstrate a lack of advance care planning and a sparse implementation of the recently published PD-specific recommendations in written ADs. To improve the current conditions, physicians should address the writing of specific ADs more frequently and earlier in the disease progress. Further, the scarce supply of palliative care training, especially for neurologists, should be extended to increase awareness for advance care planning. Therefore, the economic framework for palliative care in outpatient settings should be improved. This could lower the barriers for physicians to better support their PwP in the future and strive towards a more personalized palliative care approach.

## Figures and Tables

**Table 1:** Demographic data of the survey participants.

Variable	Answering Options	All % (*n* = 87)	GPs % (*n* = 45)	Neurologists % (*n* = 42)	Significance (Test)
Age (years)	Mean ± SD	53.5 ± 9	53,4 ± 9.5	53,5 ± 8.5	0.962 (t)
Sex	Female Male	36 (41.4%) 51 (58.6%)	19 (42.2%) 26 (57.8%)	17 (40.5%) 25 (59.5%)	0.869 (c)
Work experience (years)	Mean ± SD Missing data	25 ± 9.1 1 (1.2%)	24 ± 9.3 1 (2.2%)	26 ± 8.7 0 (0.0%)	0.250 (t)
Location of the practice	Rural Urban Missing data	53 (60.9%) 33 (37.9%) 1 (1.2%)	34 (75.6%) 10 (22.2%) 1 (2.2%)	19 (45.2%) 23 (54.8%) 0 (0.0%)	0.002 ** (c)
Number of patients per quarter	≤500 501–1000 1001–2000 >2000	14 (16.1%) 22 (25.3%) 40 (46.0%) 11 (12.6%)	2 (4.4%) 6 (13.3%) 26 (57.8%) 11 (24.4%)	12 (28.6%) 16 (38.1%) 14 (33.3%) 0 (0.0%)	<0.001 ** (c)
Special training in palliative care	Yes No	17 (19.5%) 70 (80.5%)	15 (33.3%) 30 (66.7%)	2 (4.8%) 40 (95.2%)	0.001 ** (f)

Abbreviations: PD = Parkinson’s disease; AD = advance directive; GP = general practitioner; PwP = people with Parkinson’s disease; SD = standard deviation; c = Pearson Chi-Square Test; f = Fisher’s Exact Test; t = t-test; ** *p* ≤ 0.01.

**Table 2:** Advance care planning in outpatient settings.

Section	Variable	Answering Options	All % (*n* = 87)	GPs % (*n* = 45)	Neurologists % (*n* = 42)	Significance (Test)
B	Questions about your PD patients					
B1	How many PD patients do you currently care for?	0–5 6–10 11–20 21–30 31–50 >50	19.5% (*n* = 17) 16.1% (14) 17.2% (15) 11.5% (10) 17.2% (15) 18.4% (16)	28.9%(13) 24.4% (11) 31.1% (14) 6.7% (3) 4.4% (2) 4.4% (2)	9.5% (4) 7.1% (3) 2.4% (1) 16.7% (7) 31.0% (13) 33.3% (14)	<0.001 **
B2	What percentage of your PD patients are in an advanced stage? (e.g., Hoehn and Yahr >3, complex symptoms, severe non-motor symptoms, non-oral follow-up therapies)	<25% 26–50% 51–75% >75%	46.0% (40) 39.1% (34) 13.8% (12) 1.1% (1)	60.0% (27) 33.3% (15) 6.7% (3) 0.0% (0)	31.0% (13) 45.2% (19) 21.4% (9) 2.4% (1)	<0.003 **
B3	What percentage of your PD patients also receive palliative care?	0% 1–5% 6–10% 11–20% >20% missing data	57.5% (50) 35.6% (31) 3.4% (3) 1.1% (1) 1.1% (1) 1.1% (1)	64.4% (29) 33.3% (15) 0.0% (0) 0.0% (0) 2.2% (1) 0.0% (0)	50.0% (21) 38.1% (16) 7.1% (3) 2.4% (1) 0.0% (0) 2.4% (1)	0.235

Abbreviations: PD = Parkinson’s disease; GP = general practitioner; l = Linear Trend Test; ** *p* ≤ 0.01.

**Table 3:** Supply with PD-specific advance directives in outpatient settings.

Section	Variable	Answering Options	All % (*n* = 87)	GPs % (*n* = 45)	Neurologists % (*n* = 42)	Significance (Test)
C	Questions about living wills					
C1	What percentage of your PwP have an advance directive?	<25% 26–50% 51–75% >75% do not know missing data	27.6% (24) 29.9% (26) 19.5% (17) 6.9% (6) 12.6% (11) 3.4% (3)	26.7% (12) 28.9% (13) 24.4% (11) 11.1% (5) 6.7% (3) 2.2% (1)	28.6% (12) 31.0% (13) 14.3% (6) 2.4% (1) 19.0% (8) 4.8% (2)	0.123 (l)
C2	Do you advise your PwP on the creation or modification of living wills?	yes no	65.5% (57) 34.5% (30)	75.6% (34) 24.4% (11)	54.8% (23) 45.2% (19)	0.041 * (c)
C3	How often do such counselling situations occur in a year across all PwP?	0–5 6–10 11–20 >20 missing data	77.0% (67) 11.5% (10) 5.7% (5) 4.6% (4) 1.1% (1)	84.4% (38) 6.7% (3) 4.4% (2) 4.4% (2) 0.0% (0)	69.0% (29) 16.7% (7) 7.1% (3) 4.8% (2) 2.4% (1)	0.311 (l)
C4	How long do these consultations for living wills take in total?	5–10 min 11–30 min 31–60 min >60 min several consultation dates missing data	17.2% (15) 52.9% (46) 13.8% (12) 1.1% (1) 2.3% (2) 12.6% (11)	8.9% (4) 57.8% (26) 22.2% (10) 2.2% (1) 2.2% (1) 6.7% (3)	26.2% (11) 47.6% (20) 4.8% (2) 0.0% (0) 2.4% (1) 19.0% (8)	0.018 * (l)
C5	Have you included specific aspects of PD and therapy in the living will?	yes no missing data	24.1% (21) 71.3% (62) 4.6% (4)	15.6% (7) 82.2% (37) 2.2% (1)	33.3% (14) 59.5% (25) 7.1% (3)	0.030 * (c)
C6	If yes, which PD-specific aspects have you included in the living will? (Multiple choices possible)	LCIG therapy	5.7% (5)	2.2% (1)	9.5% (4)	0.483 (f)
		Deep brain stimulation	2.3% (2)	2.2% (1)	2.4% (1)	1.000 (f)
		Nutrition and airway management for swallowing disorders	25.3% (22)	17.8% (8)	33.3% (14)	0.023 *(f)
		Catheterisation for bladder and rectal disorders	17.2% (15)	15.6% (7)	19.0% (8)	0.779 (f)
		Dementia development, personality changes	23.0% (20)	15.6% (7)	31.0% (13)	0.195 (f)
		Other	3.4% (3)	4.4% (2)	2.4% (1)	1.000 (f)
C7	Is there a note in your documentation if there is an advance directive from a PwP?	yes no missing data	60.9% (53) 37.9% (33) 1.1% (1)	77.8% (35) 22.2% (10) 0.0% (0)	42.9% (18) 54.8% 23) 2.4% (1)	0.003 ** (c)

Abbreviations: PD = Parkinson’s disease; AD = advance directive; GP = general practitioner; PwP = people with Parkinson’s disease; LCIG = Levodopa-Carbidopa intestinal gel; l = Linear Trend Test; c = Pearson Chi-Square Test; f = Fisher’s Exact Test; * *p* ≤ 0.05, ** *p* ≤ 0.01.

**Table 4:** Evaluation of the previously established PD-specific recommendations.

Section	Variable	Answering Options	All % (*n* = 87)	GPs % (*n* = 45)	Neurologists % (*n* = 42)	Significance (Test)
D	Questions about the attached recommendations					
D1	The point in time for medical discussions on PD-specific formulations about living wills should take place when the patient can safely grasp the complexity of the decisions including the consequences, seems to me...	too soon appropriate too late missing data	0.0% (0) 97.7% (85) 0.0% (0) 2.3% (2)	0.0% (0) 97.8% (44) 0.0% (0) 2.2% (1)	0.0% (0) 97.6% (41) 0.0% (0) 2.4% (1)	no testing possible
D2	How important do you find the following PD-specific recommendations for living wills?					
	Doctors should use case studies to explain specific decisions in advance directives.	important rather important rather unimportant unimportant missing data	55.2% (48) 37.9% (33) 4.6% (4) 0.0% (0) 2.3% (2)	48.9% (22) 48.9% (22) 0.0% (0) 0.0% (0) 2.2% (1)	61.9% (26) 26.2% (11) 9.5% (4) 0.0% (0) 2.4% (1)	0.775 (l)
	B. Doctors should explain the palliative medical value of a medical pump.	important rather important rather unimportant unimportant missing data	57.5% (50) 26.4% (23) 11.5% (10) 2.3% (2) 2.3% (2)	48.9% (22) 35.6% (16) 11.1% (5) 4.4% (2) 0.0% (0)	66.7% (28) 16.7% (7) 11.9% (5) 0.0% (0) 4.8% (2)	0.097 (l)
	C. Doctors should provide information on swallowing disorders and treatment options for bladder and rectal disorders in an advanced stage.	important rather important rather unimportant unimportant missing data	56.3% (49) 40.2% (35) 2.3% (2) 0.0% (0) 1.1% (1)	51.1% (23) 46.7% (21) 2.2% (1) 0.0% (0) 0.0% (0)	61.9% (26) 33.3% (14) 2.4% (1) 0.0% (0) 2.4% (1)	0.305 (l)
	D. Doctors should provide information about neuropsychiatric symptoms and their treatment early in the disease course.	important rather important rather unimportant unimportant missing data	56.3% (49) 28.7% (25) 10.3% (9) 1.1% (1) 3.4% (3)	60.0% (27) 31.1% (14) 6.7% (3) 2.2% (1) 0.0% (0)	52.4% (22) 26.2% (11) 14.3% (6) 0.0% (0) 7.1% (3)	0.464 (l)
D3	Do you care for PwP on LCIG therapy?	yes no missing data	36.8% (32) 60.9% (53) 2.3% (2)	31.1% (14) 66.7% (30) 2.2% (1)	42.9% (18) 54.8% (23) 2.4% (1)	0.251 (c)
D4	Can you imagine advising your PwP on specific aspects of LCIG therapy within the framework of an advance directive?	yes no missing data	69.0% (60) 27.6% (24) 3.4% (3)	62.2% (28) 35.6% (16) 2.2% (1)	76.2% (32) 19.0% (8) 4.8% (2)	0.097 (c)
D5	Do you care for PwP with Deep Brain Stimulation?	yes no missing data	40.2% (35) 59.8% (52) 0.0% (0)	22.2% (10) 77.8% (35) 0.0% (0)	59.5% (25) 40.5% (17) 0.0% (0)	0.001 ** (c)
D6	Can you imagine advising your PwP about specific aspects of Deep Brain Stimulation in the context of an advance directive?	yes no missing data	58.6% (51) 40.2% (35) 1.1% (1)	57.8% (26) 42.2% (19) 0.0% (0)	59.5% (25) 38.1% (16) 2.4% (1)	0.763 (c)
D7	Do you care for PwP with swallowing disorders?	yes no	86.2% (75) 13.8% (12)	75.6% (34) 24.4% (11)	97.6% (41) 2.4% (1)	0.003 ** (c)
D8	Can you imagine advising PwP on specific therapy aspects of swallowing disorders within the framework of an advance directive?	yes no	93.1% (81) 6.9% (6)	91.1% (41) 8.9% (4)	95.2% (40) 4.8% (2)	0.448 (c)
D9	Do you care for PwP with neuropsychological symptoms?	yes no	92.0% (80) 8.0% (7)	84.4% (38) 15.6% (7)	100.0% (42) 0.0% (0)	0.008 ** (c)
D10	Can you imagine advising your Parkinson’s patients on specific therapy aspects of neuropsychological symptoms within the framework of an advance directive?	yes no missing data	89.7% (78) 9.2% (8) 1.1% (1)	84.4% (38) 13.3% (6) 2.2% (1)	95.2% (40) 4.8% (2) 0.0% (0)	0.157 (c)
D11	Can these recommendations be implemented in when counselling PwP regarding living wills?	yes no missing data	79.3% (69) 17.2% (15) 3.4% (3)	86.7% (39) 13.3% (6) 0.0% (0)	71.4% (30) 21.4% (9) 7.1% (3)	0.222 (c)
D12	Who should advise on PD-specific aspects in an advance directive?	GP Neurologists both other missing data	3.4% (3) 23.0% (20) 67.8% (59) 4.6% (4) 1.1% (1)	4.4% (2) 22.2% (10) 71.1% (32) 2.2% (1) 0.0% (0)	2.4% (1) 23.8% (10) 64.3% (27) 7.1% (3) 2.4% (1)	0.614 (c)

Differences to 100% in the sum of the descriptive values are possible due to rounding. Abbreviations: PD = Parkinson’s disease; AD = advance directive; GP = general practitioner; PwP = people with Parkinson’s disease; LCIG = Levodopa-Carbidopa intestinal gel; l = Linear Trend Test; c = Pearson Chi-Square Test; ** *p* ≤ 0.01.
